# Using Dental Enamel Wrinkling to Define Sauropod Tooth Morphotypes from the Cañadón Asfalto Formation, Patagonia, Argentina

**DOI:** 10.1371/journal.pone.0118100

**Published:** 2015-02-18

**Authors:** Femke M. Holwerda, Diego Pol, Oliver W. M. Rauhut

**Affiliations:** 1 Staatliche Naturwissenschaftliche Sammlungen Bayerns (SNSB), Bayerische Staatssammlung für Paläontologie und Geologie, München, Germany; 2 GeoBioTec, Departamento de Ciências da Terra, Faculdade de Ciências e Tecnologia (FCT), Universidade Nova de Lisboa, Caparica, Portugal; 3 Department of Earth and Environmental Sciences and GeoBioCenter, Ludwig Maximilians Universität, München, Germany; 4 Consejo Nacional de Investigaciones Científicas y Técnicas (CONICET), Buenos Aires, Argentina; 5 Museo Paleontológico Egidio Feruglio, Trelew, Argentina; University of Pennsylvania, UNITED STATES

## Abstract

The early Middle Jurassic is regarded as the period when sauropods diversified and became major components of the terrestrial ecosystems. Not many sites yield sauropod material of this time; however, both cranial and postcranial material of eusauropods have been found in the Cañadón Asfalto Formation (latest Early Jurassic–early Middle Jurassic) in Central Patagonia (Argentina), which may help to shed light on the early evolution of eusauropods. These eusauropod remains include teeth associated with cranial and mandibular material as well as isolated teeth found at different localities. In this study, an assemblage of sauropod teeth from the Cañadón Asfalto Formation found in four different localities in the area of Cerro Condor (Chubut, Argentina) is used as a mean of assessing sauropod species diversity at these sites. By using dental enamel wrinkling, primarily based on the shape and orientation of grooves and crests of this wrinkling, we define and describe three different morphotypes. With the exception of one taxon, for which no cranial material is currently known, these morphotypes match the local eusauropod diversity as assessed based on postcranial material. Morphotype I is tentatively assigned to Patagosaurus, whereas morphotypes II and III correspond to new taxa, which are also distinguished by associated postcranial material. This study thus shows that enamel wrinkling can be used as a tool in assessing sauropod diversity.

## Introduction

Sauropods are one of the most successful groups of dinosaurs; their presence in the fossil record stretches from the Late Triassic to the Late Cretaceous (e.g. [1–4]). Among them, the eusauropods diversified and became abundant in terrestrial ecosystems during the Jurassic period, and representatives of most important lineages of this clade gained a global distribution. Phylogenetic studies indicate that especially the Middle Jurassic was an important time in eusauropod evolution, with a proliferation of basal forms and the first appearance of neosauropods, including many of the lineages that radiated in the Late Jurassic and Cretaceous [5,6]. Nevertheless, Middle Jurassic dinosaur localities are relatively rare [7], with most material coming from China e.g. [8–12]. Apart from the Chinese record, a few taxa are known from northern Africa [13–16], Madagascar [17,18] and Argentina [19–26].

Most of the Jurassic eusauropod remains discovered in Argentina come from the Cañadón Asfalto Formation [27], which crops out in north-central Chubut Province, Argentinean Patagonia, and has recently been dated as ranging from the latest Early to the early Middle Jurassic (Toarcian to Aalenian-Bajocian; [28]). Vertebrate fossils are very abundant in this unit and include anurans [29], lepidosaurs [30], turtles [31,32], mammals [33–37], pterosaurs [38,39], and ornithischian [40–42], theropod (e.g. [19,20,43,44]), and eusauropod dinosaurs [19,21,25], contributing to a relatively complete Middle Jurassic ecosystem [45]. Eusauropod dinosaurs have so far mainly been reported from bonebeds and include *Patagosaurus fariasi* Bonaparte 1979 [19], *Volkheimeria patagonicus* Bonaparte 1979 [19], and at least two undescribed taxa [22,23,25]. *Patagosaurus* is known from several individuals with both cranial and postcranial elements represented [19,21], whereas *Volkheimeria* is exclusively known from postcranial material, presumably from a single individual [19]. The two undescribed taxa are known from both postcranial and cranial material. One of them was originally referred to *Patagosaurus* by Bonaparte [19,21]; however, serious doubt on the validity of this assignment exists due to significant differences between material of the holotype of *Patagosaurus fariasi* and the other collected material [23,46]. The fourth eusauropod taxon has been found at the base of the unit and differs from the three other specimens in its postcranial and cranial anatomy [25].

**Fig 1:**
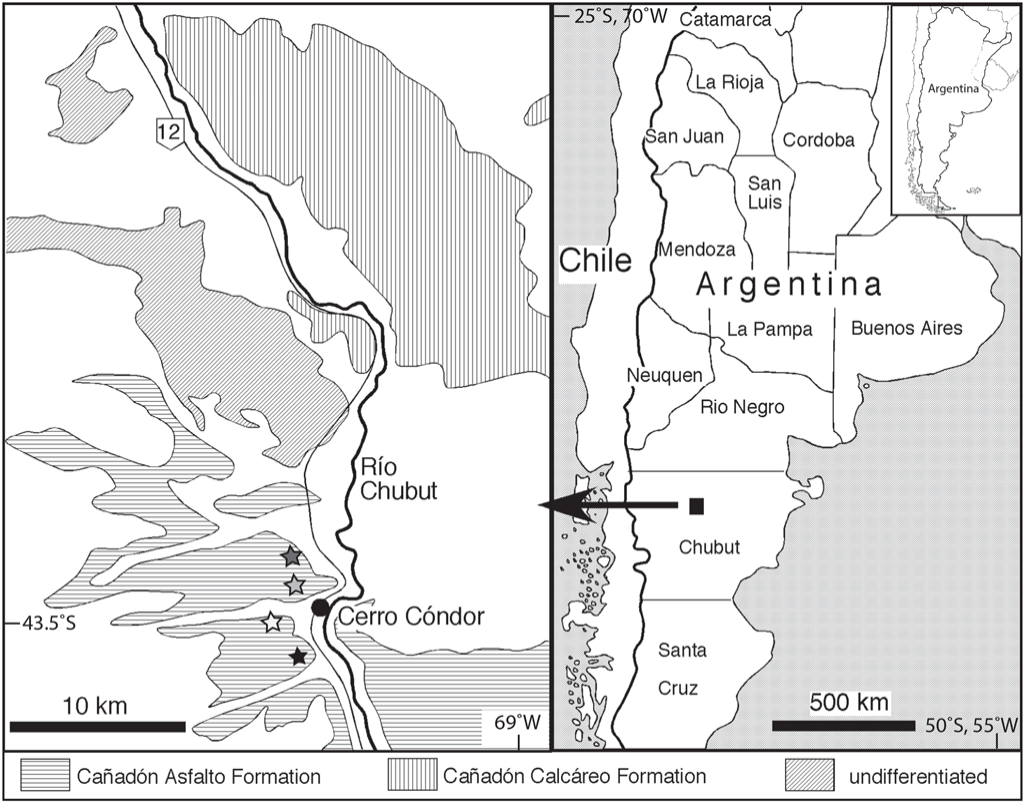
Cerro Condor Norte (dark grey asterisk) Sur (light grey asterisk), Las Chacritas (white asterisk) and Bagual (black asterisk) localities in Chubut, Argentina (modified from Rauhut [94]).

Most material comes from the locality of Cerro Condor Sur in the Cañadón Asfalto Formation (Fig. 1). This locality is the major bonebed discovered by Bonaparte [19,20] and has yielded remains of eusauropods referred to *Patagosaurus* and *Volkheimeria*, and the basal tetanuran theropod *Piatnitzkysaurus*, representing the most fossiliferous site for the Jurassic of South America. Several isolated sauropod teeth have been recovered at this site. Until recently, isolated fossil teeth were considered non-diagnostic taxonomically. Many studies however have used isolated theropod teeth as a means to measure species diversity, even if a secure referral to known taxa is not possible, and morphological variation along the toothrow has been documented in theropods (e.g. [47–55]).

Sauropod teeth have recently been used to recognize the presence of certain clades [56–58] or to assess species diversity [59][60]. Certain characters are frequently used for taxonomic purposes or as phylogenetic characters, such as tooth shape/elongation, presence/absence of denticles and grooves, wear facet shape and orientation, and enamel wrinkling pattern [3,61,62]. Enamel wrinkling has been retrieved as a synapomorphy (related to herbivorous specialization) in Eusauropoda [63], although some basal sauropodomorphs also had incipient wrinkling on the enamel (e.g. [64]). Tooth shape varies among major clades of eusauropods, such as basal neosauropods, diplodocoids, and titanosaurs [1,61,62], allowing high-level taxonomic classification. The orientation of the wear facet, reflecting the position of teeth during occlusion relative to other teeth, also generally differs among clades (although intraspecific differences in wear facets exist)[65–68].

In some sauropod descriptions specific tooth characters are noted as characteristic of a particular species or higher taxon, albeit they have not always been explicitly presented as autapomorphies. For instance, the lingual crown buttress has been regarded as autapomorphic for *Euhelopus* [69], (although recently found in the new titanosaur *Yongjinglong* [70]) and the asymmetry of mesial and distal denticles has been noted as characteristic for *Omeisaurus* [9] (Tang et al. 2001, however this is also found in unerrupted teeth of *Bellusaurus*; [71]). The crowns of *Shunosaurus* are notably slender but spoon-shaped as noted in the diagnosis [72]. The presence of two types of wear (apical and V-shaped) has been noted as a unique feature of *Nemegtosaurus* [73], but has later been reported from the closely related *Tapuiasaurus* as well [74]. Diplodocids are characterized by asymmetrical enamel wrinkling distribution, and Rebbachisaurids have extreme asymmetrical enamel distribution [75], which has not been found in other sauropods. In some cases characteristics of the enamel wrinkling pattern have been explicitly regarded as autapomorphic at the species level (e.g., *Amygdalodon*, [26]; *Chebsaurus*, [15]). In other cases, peculiarities on the enamel wrinkling patterns have been noted for several taxa, such as very low, rounded, occasionally anastomosing undulations along the length of the tooth for *Alamosaurus* [76], characteristic reticulate wrinkling in *Euhelopus* [69], and enamel that is finely wrinkled throughout the crown but arranged into coarser longitudinal ridges in *Nemegtosaurus* [73], although these features of the enamel wrinkling pattern have not been explicitly treated as autapomorphies.

The aim of this study is to present a morphological analysis of eusauropod teeth from localities within the Cañadón Asfalto Formation, in order to estimate eusauropod species diversity at these localities. This will aid ongoing research on eusauropod species diversity from the Cañadón Asfalto Formation (including a revision of material currently referred to *Patagosaurus fariasi*).

### Geological setting

The tooth specimens were found in the Cerro Condor area, Chubut province, Patagonia, Argentina (Fig. 1) in outcrops of the Cañadón Asfalto Formation, a continental unit consisting of mainly lacustrine deposits. In the area of Cerro Condor, the outcrops of the Cañadón Asfalto Formation are dominated by microbial limestones, often tuffaceous mudstones and shales with conchostracans, and conglomeratic intercalations [27,77]. The specimens used in this study were found in four localities; Cerro Condor Norte, Cerro Condor Sur, Las Chacritas and Bagual (Fig. 1). All of these localities represent bonebeds that are dominated by sauropod remains. The Cerro Condor Norte and Cerro Condor Sur localities have originally been excavated and described by Bonaparte [19,20], although additional material from the latter locality has been recovered subsequently (e.g. [23]). Bonaparte [20] described the geological setting of Cerro Condor Norte and Cerro Condor Sur as finely layered calcareous tuff and arenaceous tuff, respectively. He interpreted these sediments as floodplain deposits, but the general geological setting makes an interpretation as lacustrine deposits more likely. Dinosaur remains occur as mainly disarticulated elements in both localities, although some articulated elements are present (Bonaparte [20], P. Puerta, pers. com. to OR, 2000). Our observations (by DP and OR) at the locality Cerro Condor Sur indicate that the fossiliferous layer is a conglomeratic intercalartion in lacustrine deposits. The age of the Cañadón Asfalto Formation has long been considered to be Callovian-Oxfordian, but recent dates, both radiometric [78,79] and biostratigraphic [80,81] indicate a considerably older, early Middle Jurassic (Aalenian—earliest Bathonian) age for this unit, and a recent radiometric date from the base of the formation even yielded a latest Early Jurassic (Toarcian) age [28]. The Cañadón Asfalto Formation has undergone intense tectonic deformation, both folding and faulting, which makes a correlation of the different localities impossible at the moment.

## Materials and Methods

No permits were required for the described study, which complied with all relevant regulations. See Table 1 for all relevant Argentine collection references, see discussion for other references.

**Table 1:** Tooth and craniomandibular specimens used for this study, affiliations and localities.

Specimen	Locality	Material	Referred taxon
MACN-CH 2008.1	Cerro Condor Sur	isolated tooth	-
MACN-CH 2008.2	Cerro Condor Sur	isolated tooth	-
MACN-CH 2008.3	Cerro Condor Sur	isolated tooth	-
MACN-CH 2009	Cerro Condor Sur	isolated tooth	-
MACN-CH 934	Cerro Condor Sur	maxilla	*Patagosaurus* *
MPEF-PV 1670	Cerro Condor Sur	dentary	*Patagosaurus* **
PVL 4076	Cerro Condor Sur	premaxila	*Patagosaurus* *
MACN-CH 933	Cerro Condor Norte	dentary	*Patagosaurus* *
MPEF-PV 3058	Las Chacritas	isolated tooth	-
MPEF-PV 3059	Las Chacritas	isolated tooth	-
MPEF-PV 3060	Las Chacritas	isolated tooth	-
MPEF-PV 3061	Las Chacritas	isolated tooth	-
MPEF-PV 3341	El Bagual	maxilla	-

*[21]

**[23]

Materials: the fossil material used for this study includes isolated sauropod teeth as well as craniomandibular material associated with teeth. There are four isolated teeth from Cerro Condor Sur, and four from the Las Chacritas locality (Table 1). Craniomandibular material includes a juvenile dentary MACN-CH 933 (one tooth used for this study) from Cerro Condor Norte. This dentary is referred to *Patagosaurus* by Bonaparte (1986) by overlap of associated postcranial material with that of the holotype [23] (Table 1). Further material used here is one premaxilla PVL 4076 (one only partially exposed, not-measurable tooth used) from Cerro Condor Sur, which was described and referred to *Patagosaurus* by Bonaparte [21], a maxilla MACN-CH 934 (12 alveoli, 2 teeth used of which one only partially exposed) from Cerro Condor Sur, also referred to *Patagosaurus* by Bonaparte [21], and a maxilla MPEF-PV 3341 with associated dentition from the El Bagual locality (reported by [25]). Finally a dentary MPEF-PV 1670 (two teeth used for this study) from Cerro Condor Sur, found to be similar to MACN-CH 933 (therefore assigned to *Patagosaurus*, albeit in a later ontogenetic state, [23]) was examined. See geological setting for the localities.

The teeth were gently cleaned using a toothbrush and acetone. To obtain better images of the enamel wrinkling pattern teeth were coated with magnesium oxide.

Imaging: the fossil teeth were photographed with and without magnesium coating with a Nikon stereoscope in high resolution. Also, SEM pictures were taken at the microscopy lab at Aluar Aluminio Argentino (Puerto Madryn, Chubut, Argentina). For this the specimens were gently cleaned but not coated with any material.

Terminology and morphological characters: tooth orientation follows Smith and Dodson [82]. To define tooth morphology, we measured the Slenderness Index (SI), which was determined for each tooth by dividing the apicobasal length by its mesiodistal width [61], and we determined the presence or absence of lingual/labial grooves. Tooth enamel wrinkling pattern was studied at the base, middle, and apex of the crown. Labial and lingual wrinkling patterns were found to be similar. Especially the enamel wrinkling pattern at the base of the crown was taken into consideration during analysis, since wear can have profound effects on the wrinkling at the apex of the teeth (see for instance *Nemegtosaurus* teeth by [73]). Apical wear facets and denticles were studied, if present. Wear facets were studied in respect to their position and size. Denticle density was obtained by dividing number of denticles by the mesiodistal maximum width (in mm) of the denticle-bearing tooth crown.

For the description of dental enamel wrinkling, we used the following terminology; ‘grooves’ are longitudinal (apicobasally oriented) depressions on the enamel surface, whereas ‘crests’ are protruding continuous structures of the enamel. ‘Sulci’ are circular depressions surrounding ‘islets’, rounded discontinuous protrusions.

## Results

### General morphology

**Fig 2:**
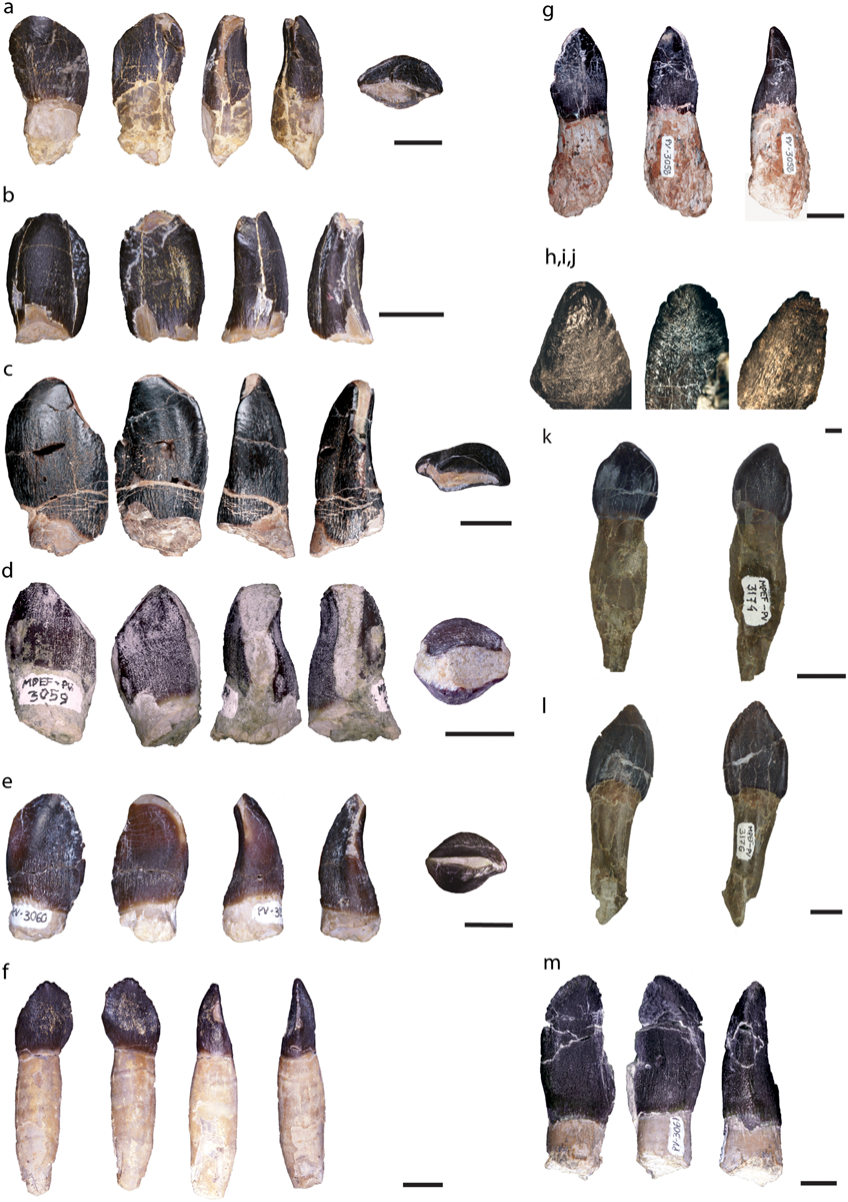
General morphology of teeth shown in labial, lingual, and (where possible) in lateral and apical views, a: MACN-CH 2008.1; b: MACN-CH 2008.2; c: MACN-CH 2008.3; d: MPEF-PV 3059; e: MPEF-PV 3060; f: MACN-CH 2009; g: MPEF-PV 3058; h: MACN-CH 934; i: MPEF-PV 1670; j: MACN-CH 933; k,l: MPEF-PV 3174 and 3176 belonging to maxilla MPEF-PV 3341; m: MPEF-PV 3061.

All isolated teeth have a convex labial side and concave lingual surface, creating a D-shaped cross section (Fig. 2). The crowns are spatulate in lingual and labial view, a feature commonly found in basal eusauropods (“Cetiosaurids, *Shunosaurus*-type sauropods and higher eusauropods” sensu [1]). All teeth exhibit wrinkling, which is an autapomorphy for Eusauropoda [61,62]. Most teeth have wear facets, which are evidence of dental occlusion, also a feature common for eusauropods [61,62] (Fig. 2).

The SI values range between 1.1 (MPEF-PV 3341) and 1.9 (MPEF-PV 3061) with a mean of 1.38 and a standard deviation of 0.37, except for the only measurable dentary tooth MPEF-PV 1670 that has an SI value of 2.6 (Table 2). These values place all teeth within the ‘Broad-Crowned’ (BC) tooth category (SI < 4.0) defined by Barrett & Upchurch [1]. Similarly, plotting the log SI values vs. age scatterplot, as done by Chure et al. [83], places these teeth in the broad range of ‘basal sauropods’ (using the age constraint of [28]).

**Table 2:** General tooth measurements and characteristics.

Specimen	SI	Position	Grooves lingual		Grooves labial		Denticle density	Position wearfacet	Transverse shape
			distal	mesial	distal	mesial			
MACN-CH 2008.1	1,2	left dentary or right maxilla	1	1	0	1	0	distal	D
MACN-CH 2008.2	1,5	right maxilla or left dentary	1	1	1	1	0	(broken)	D
MACN-CH 2008.3	1,7	left dentary	1	1	1	0	0	mesial and distal	D
MACN-CH 2009	1,4	left maxilla or right dentary	0	0	0	0	0,7	(unworn)	D
MACN-CH 933	?	dentary	?	?	?	?	1,1	(unworn)	?
MACN-CH 934	?	one left maxilla	?	?	?	?	0	(unworn)	?
MPEF-PV 3058	1,5	right dentary or left maxilla	0	1	1	1	0	(broken)	D
MPEF-PV 3059	1,4	left dentary	0	0	1	1	0	mesial and distal	D
MPEF-PV 3060	1,5	right maxilla	0	1	1	0	0	mesial	D
MPEF-PV 3061	1,9	right dentary or left maxilla	1	1	1	0	0,7	(unworn)	D
MPEF-PV 1670	2,6	dentary	?	?	?	?	0,8	(unworn)	?
MPEF-PV 3341 1	1,1	left maxilla	1	0	1	0	0	broken	?
MPEF-PV 3341 2	0	left maxilla	0	0	0/1	0/1	0	worn away	?
MPEF-PV 3341 3	1,2	left maxilla	0	0	1	0	0	mesial and distal??	?
MPEF-PV 3341 4	1,2	left maxilla	0	1	1	0	1,3	-	D
MPEF-PV 3341 5	0	left maxilla	0	0	0	0	0	-	D
MPEF-PV 3341 6	1,4	left maxilla	0/1	0/1	1	0	0	mesial	D
MPEF-PV 3341 7	0	left maxilla	0	0	0	0	0	mesial and distal?	D
MPEF-PV 3341 8	0	left maxilla	0	0	0	0	0	mesial and distal?	?
MPEF-PV 3341 9	1,3	left maxilla	1	0	1	0	0	mesial and distal	D
MPEF-PV 3341 10	1,2	left maxilla	0	0	0	0	0	mesial	D
MPEF-PV 3341 11	1,3	left maxilla	1	1	1	0	0	mesial	D

All teeth (except for MACN-CH 2009) possess apicobasal grooves either on their lingual or labial surface, but their presence along the distal or mesial margin varies greatly (Table 2). Only one tooth (MACN-CH 2008.2), possesses all four grooves, all other teeth lack at least one of the crown grooves. Some teeth have incipient and very shallow grooves (e.g., distal-lingual in MACN-CH 933 and mesio-lingual in MPEF-PV 1670, Table 2).

Five specimens have denticles preserved (Table 2), all of which are restricted to the apical region of the crown, extending along its mesial and distal margins (between ~10–20% of the crown height). The denticle density ranges from 0.7 to 1.3, which does not differ much between specimens. The denticles of MACN-CH 2009 and MPEF-PV 3341 (4) are asymmetrically distributed on the apex; in MACN-CH 2009 there are 2–3 denticles on the apical mesial side and 3–4 on the apical distal side, in MPEF-PV 3341 (4) these numbers are 8 for the mesial and 2–3 for the distal apical side. MPEF 1670 and MACN-CH 933 have denticles that are more symmetrically distributed on the apex, with one larger denticle at the apex and 2–3 denticles along the distal and mesial margins. MPEF-PV 3061 has a similar symmetrical denticle distribution, however with 4 denticles on the apico-distal side and 3–4 apico-mesially (though the mesial part is broken).

All worn teeth have V-shaped wear facets, similar to those of the reconstruction of wear from occlusion in *Camarasaurus [65]*. The position of wear facets varies, given that some teeth have both a mesial and distal wear facet and others only have one well-developed mesial or distal facet.

As shown above, the high (and usually continuous) variability of several aspects (e.g., Slenderness Index (SI), crown shape, labial/lingual grooves, denticle density), as well as their variation along the toothrow in other eusauropods [61,63,75,84] precludes the use of these features to define morphotypes for taxonomic purposes. For instance, tooth shape and size may vary between maxillary and dentary teeth, as noted for *Shunosaurus* [10], *Abydosaurus* [83], and *Nemegtosaurus* [73]. Additionally, the presence of grooves can also vary between the upper and lower toothrow and this might explain the lack of a clear pattern in the presence/absence of labial/lingual grooves. For instance, both mesial and distal grooves seem to be present lingually, and only distal grooves labially in the toothrow of the dentary in *Camarasaurus* (CM 11338). In the maxilla, the distal groove seems to be present on the labial but not the mesial side (lingual side not visible). In a toothrow of *Giraffatitan* (MB.R. 2181.23.9), on the lingual side the mesial and in some teeth also the distal groove is visible, but not in all; labially the distal groove is present in most teeth; the mesial groove is not present in most teeth. Finally, the teeth in the maxilla MPEF-PV 3341 from the Bagual locality also show variation in the development of grooves along the toothrow (Table 2).

Among the analyzed teeth from Cañadón Asfalto there is marked variation in the development and size of the wear facets. High tooth replacement rates and extensive wear facets also affect many of the above mentioned features of the teeth [75], including crown shape, presence of denticles, height of the crown (and therefore SI), and, as has recently been noted, *Patagosaurus* had a moderately high tooth replacement rate of 58 days [75]. Finally, denticle density has not been used primarily because it is only comparable between newly erupted (unworn) teeth.

### Enamel wrinkling

**Fig 3:**
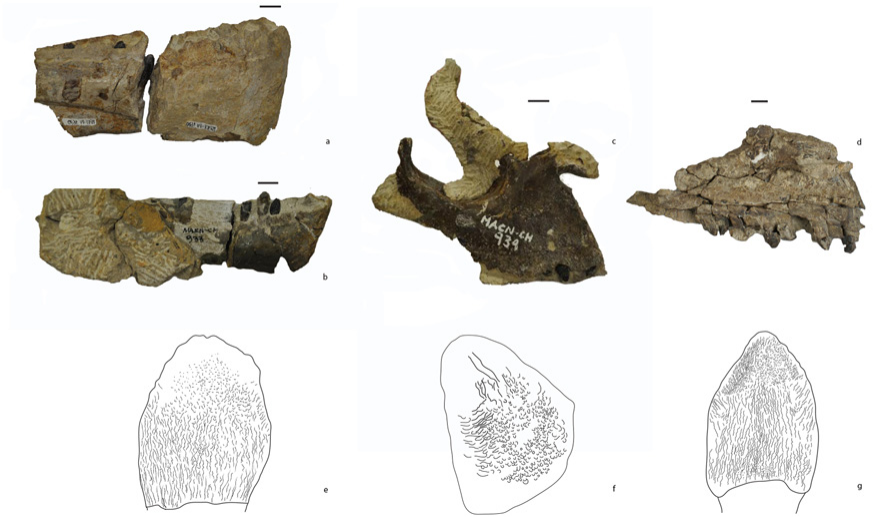
Craniomandibular material from the Cañadón Asfalto Formation, with tooth morphotypes based on enamel wrinkling. a: MPEF-PV 1670; b: MACN-CH 933; c: MACN-CH 934; d: MPEF-PV 3341; e: Morphotype I; f: Morphotype II; g: Morphotype III.

Among the four (partial) toothrows (Fig. 3a, b, c, d) preserved in the analyzed sample from the Cañadón Asfalto Formation, the enamel wrinkling pattern seems to be unaffected by the position of the teeth along the toothrow and is mostly unaffected by tooth wear, especially at the base of the crown. This is probably true for other eusauropods and higher sauropods; however, in most descriptions the enamel wrinkling (and its variation along the toothrow) has not been studied in detail. An exception to this is the description by Wilson [73] of *Nemegtosaurus*, who notes that although tooth shape changes along the toothrow, and between maxilla and dentary teeth, the enamel wrinkling (especially at the base) remains the same. A complete maxillary toothrow of the material analyzed here (MPEF-PV 3341) includes teeth in various stages of eruption (and thus in wear) but the enamel wrinkling pattern at the base of the crown does not vary. Furthermore, in specimens of *Camarasaurus* (CM 11338) and *Giraffatitan* (MB.R.2181.23.9) with complete toothrows preserved the pattern of enamel wrinkling at the base of the crown does not seem to vary along the toothrow.

Among the analyzed sample there are three different types of enamel wrinkling (when the base of the crown is taken into consideration, Fig. 3e, f, g; see morphotypes). For each type, the wrinkling at the base of the crown has not been affected by the degree of wear of the tooth (newly erupted, unworn, or worn). Therefore, in this section we define three different morphotypes for the sample of teeth from the Cañadón Asfalto Formation using enamel wrinkling as the primary character.

### Morphotype I

**Characteristics**

**Fig 4:**
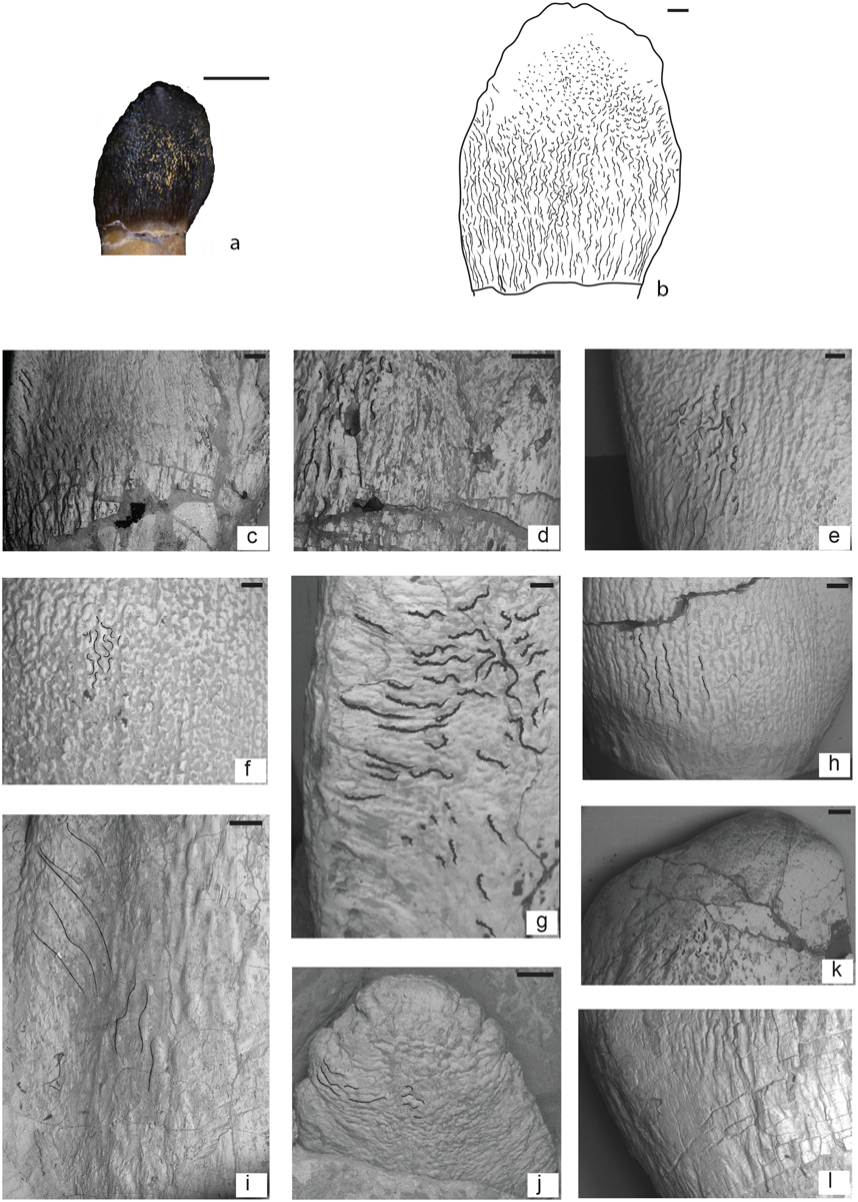
Morphotype I: a: exemplary specimen MACN-CH 2009 with b: schematic drawing of wrinkling pattern. c: MACN-CH 2008.1; d: MACN-CH 2008.3; e: MPEF-PV 3061; f: MPEF-PV 3059; g: MPEF-PV 1670; h: MPEF-PV 3060; i: MACN-CH 933; j: MPEF-PV 1670 newly erupted tooth; k: MACN-CH 2008.2; l: MPEF-PV 3058.

Wrinkling pattern at base of tooth composed of apicobasally oriented crests and grooves (Fig. 4b, c, d, e, h, l). These crests can be continuous and subparallel to each other, or sinuous and interrupted by grooves. Towards the apex the wrinkling pattern is highly sinuous, with islets, pits, and sulci (indentations; Fig. 4b, j, k). Most of the studied specimens belong to morphotype I: MACN-CH 2008.1, MACN-CH 2008.2, MACN-CH 2008.3, MACN-CH 2009, MACN-CH 933, MPEF-PV 3061, MPEF-PV 3060, MPEF 3059, MPEF-PV 3058 MPEF-PV 1670 and possibly PVL 4076. The shape of these teeth is not uniform (See Fig. 2).

**Description**

Base of the crown: The enamel wrinkling pattern consists of apicobasally oriented crests and grooves. Within this morphotype there is some variation on how straight or continuous the crests and grooves at the base of the crown are. Some teeth have mostly straight and subparallel crests (MACN-CH 2008.1,2,3; MPEF-PV 3058; MPEF-PV 1670; MACN-CH 933; Fig. 2 and Fig. 4c, d, i, l) whereas in others the crests are sinuous and frequently interrupted by grooves or pits (MPEF-PV 3059; 3060, 3061; Fig. 2 and Fig. 4e, f, h).

Middle to apex of the crown: Towards the apex the crests and grooves are not apicobasally elongated but become rather short, highly sinuous, and deflect towards the mesial and distal margins of the crown (Fig. 4b, c, g, j). Apically the wrinkling smoothens and forms rounded protrusions (islets) surrounded by sulci (even in unerupted and/or unworn teeth; e.g., MACN-CH 933, MPEF-PV 1670; Fig. 4g, i, j). The sulci and grooves are shallower at the apical region than at the base of the crown, a pattern also seen in unerupted and/or unworn teeth (MACN-CH 933 and MPEF-PV 1670). Towards the carinae the sinuous grooves and crests are set nearly perpendicular to the apicobasal axis of the crown (Fig. 4b, g, i).

The teeth included in this morphotype have varying stages of wear and this alters the wrinkling pattern at the middle to apex of the crown. MPEF-PV 3059 is extremely worn and has only very shallow sinusoid wrinkling preserved (Fig. 4f), whereas MPEF-PV 3060 is less worn and the sinuous crests and grooves are more pronounced (Fig. 4h). Although there is some variability in the wrinkling patterns within the teeth included in morphotype I, they can still be referred to a single type due to their similar pattern at the base of the crown.

Denticles are visible in some of the teeth (e.g., MACN-CH 933, MACN-CH 2009, MPEF-PV 3061). The absence of denticles in other teeth could be due to wear. These denticles are located along the uppermost apical parts of both the mesial and distal carina of the crown and the denticle density ranges from 2.5 (MPEF-PV 3061) to 3.64 (MACN-CH 933). The SI of these teeth ranges between 1.2 to 2.6.

### Morphotype II

**Characteristics**

Smooth or very subtly wrinkled enamel at the base, with a pebbly enamel pattern equally distributed on the middle and apex of the tooth. Only the teeth of one specimen, MACN-CH 934 belong to this morphotype, and the SI of the teeth in this maxilla could not be measured.

**Description**

**Fig 5:**
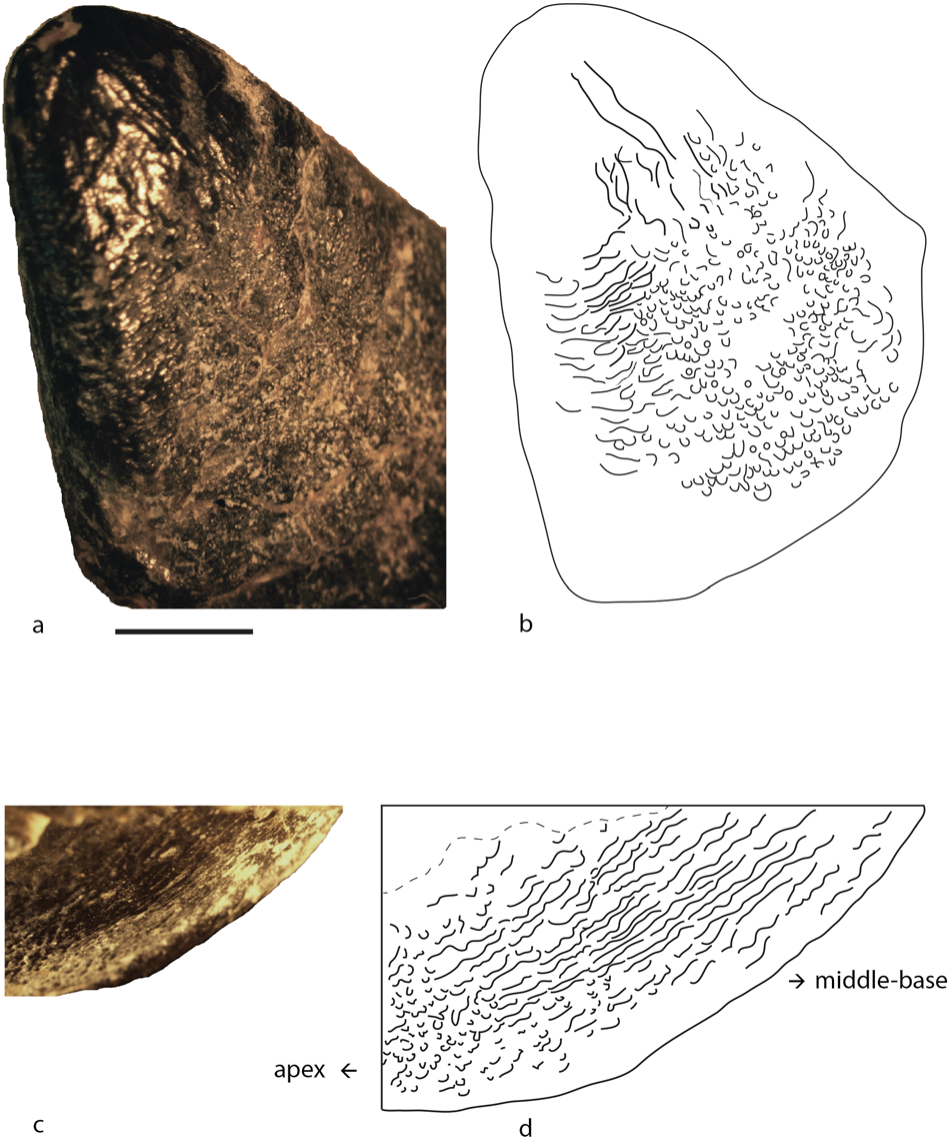
Morphotype II: a: lingually exposed surface of tooth MACN-CH 934, b: Schematic drawing of wrinkling pattern; c: Labially exposed surface of replacement tooth MACN-CH 934 (note that the maxilla prevents a better view); d: Schematic drawing of (exposed) wrinkling pattern.

Base of the crown: morphotype II displays a pebbly, ‘bubbly’ pattern, which is more pronounced in the two lingual grooves (Fig. 5a, b). The wrinkling is formed by pebbly protrusions, which are irregularly shaped (Fig. 5a, b). This pattern is more pronounced lingually than labially. Labially the tooth has subtle apicobasal wrinkling, until the pebbly pattern emerges towards the apex (Fig. 5c, d). Lingually the pebbly pattern persists up to the apex where it smoothens into subtle wrinkling again. Towards the carinae the wrinkling spreads out into shallow wrinkling grooves. No denticles are present, even though the teeth display little to no wear.

### Morphotype III

**Characteristics**

**Fig 6:**
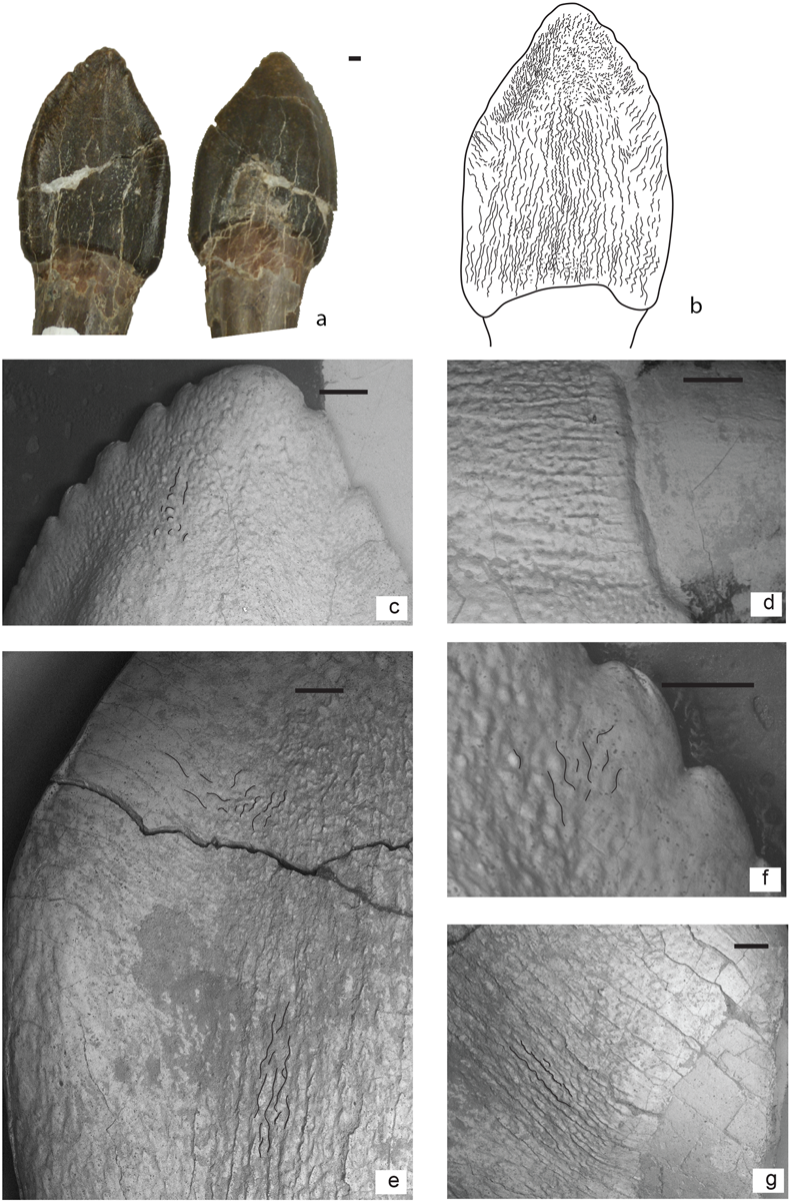
Morphotype III: a: MPEF-PV 3341 maxillary tooth lingual and labial view; b: Schematic wrinkling; c: Apical part with denticles; d: Characteristic subcircular pits; e: Carina; f: Denticles in higher magnification; g: Bulge at base.

Well-developed, smooth enamel bulge at the base of the crown that creates a sharp limit from the base of the crown to the root (Fig. 6d, g). Above the basal enamel bulge, the enamel has a wrinkling pattern composed of subcircular pits that cover most of the base of the crown, and a few apicobasal grooves (Fig. 6d, e, g). At the middle of the crown this type of wrinkling persists in unworn specimens and consists of more irregular shaped crests and pits in worn specimens. At the apex the wrinkling shows small round protrusions and pits.

Unworn teeth of this morphotype have well-developed and acute denticles along their mesial and distal margins (Fig. 6.c, f). The teeth clustered in this morphotype belong to a maxillary toothrow, MPEF-PV 3341, and show various stages of wear. The SI of the teeth from this maxilla ranges between 1.1 and 1.4, with an average of 1.25 and a standard deviation of 0.11.

**Description**

Base of the crown: The base of the crown has a characteristic bulge where enamel separates the crown from the root (Fig. 6.d, g). The wrinkling at the base of the crown consists of apicobasally directed, finely wrinkled but well-defined continuous crests and subcircular pits (Fig. 6.d, e, g). This type of wrinkling is also present at the carinae at the base, however with a slight deflection towards the carinae of about 20°.

Middle-apex of the crown: At the middle of the tooth the previously continuous crests of the wrinkling become more discontinuous, resulting in apicobasally distributed reticulate crests and grooves. These grooves and crests are visible until up to about 60% of the apicobasal height of the crown. Towards the apex the wrinkling consists of pits and small subcircular protrusions that are well separated from each other (Fig. 6.c).

Towards the carinae, at the base to up to about 50% of the crown height, the wrinkling is similar to the wrinkling at the base, however with more pronounced grooves (Fig. 6.e). At the apical part of the crown, the wrinkling deflects towards the carinae at 45° (Fig. 6.e). In worn specimens, the wrinkling at the apical part of the carinae is smooth and does not show much wrinkling. In unworn specimens, the wrinkling deflects towards the denticles as round protrusions (Fig. 6.c, f). In unworn specimens this wrinkling persist up to about 60% of the length of the denticles.

## Discussion

### Morphological & ontogenetic variation

Morphotype II and III are only found in the maxillae MACN-CH 934 and MPEF-PV 3341, respectively. Morphotype I comprises all isolated teeth, together with those found in the two dentaries (MACN-CH 933, MPEF-PV 1670), covering a broad range of shapes and sizes.

Differing shapes and size may be explained by serial variation among the toothrow as stated before, or ontogenetic variation. Rauhut et al. [85] found that in theropods, ontogenetic variation may be a factor that should be taken into account while analyzing tooth morphology. In sauropods, few examples are known of preserved ontogenetic stages of the dentition; so far these are specimens of *Camarasaurus* (one possible *Camarasaurus* embryo Britt and Naylor [86] and a subadult, CM 11338), diplodocid (juvenile SMA N29–2 and adult CM 11161), and post-hatchling, juvenile and adult specimens from the basal sauropodomorph *Mussaurus* [64,87], as well as different ontogenetic stages of isolated teeth assigned to the titanosaurian *Lirainosaurus* [59,88]. Furthermore, the embryonic titanosaurian sauropods from the Auca Mahuevo nesting site have well-preserved teeth, which can be compared to those of adult titanosaurians [89,90].

**Fig 7:**
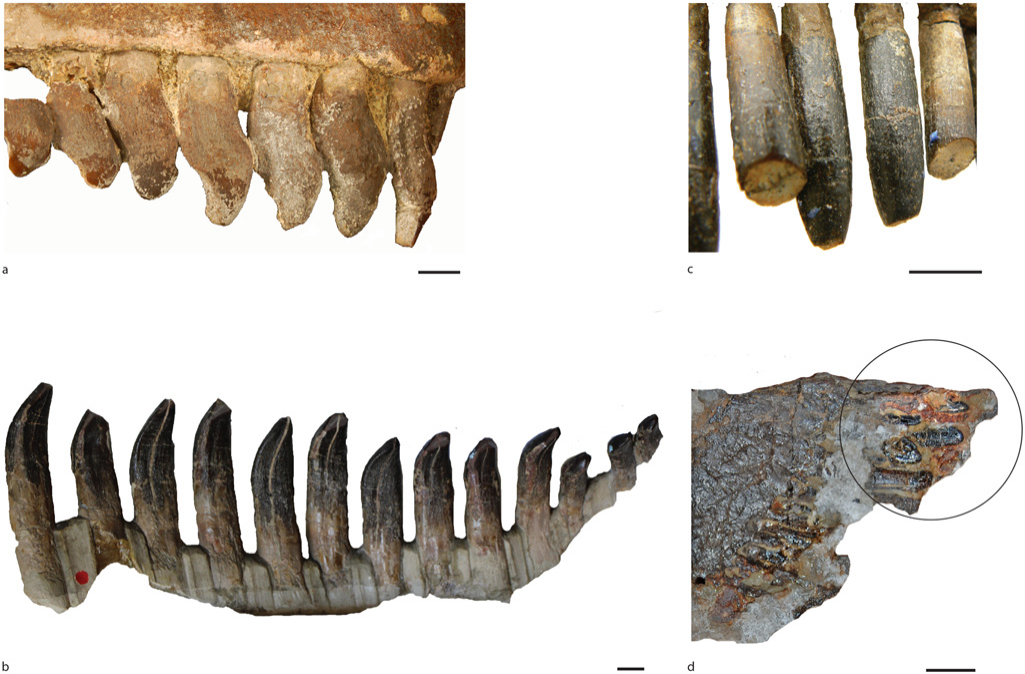
Toothrows for comparison a: Camarasaurus CM 11338; b: Giraffatitan MB.R.2181.23.9; c: Diplodocid CM 11161; d: Juvenile diplodocid SMA N29–2.

In the basal sauropodomorph *Mussaurus* there are no notable differences in dentition between post-hatchlings and juvenile specimens [64] but the adult condition is still unknown. The teeth within the possible embryonic *Camarasaurus* premaxilla are described as ‘robust, spoon-shaped and curve strongly lingually’ ([86], p. 260 Fig. 16.4); however, they appear to be more slender than subadult and adult *Camarasaurus* teeth, which are described as spoon-shaped [3,67,91]. The enamel wrinkling is described as rugose, and is the same as in the adult state. In diplodocids, although tooth shape is generally similar, small differences are found between the dentition of juveniles and adults [92]. The juvenile described (CM 11255) has upper teeth that are mesiodistally asymmetric and distally inclined in relation to those of adult specimens [92]. However, in undescribed juvenile specimens of possible diplodocids (SMA N29–2) the enamel wrinkling seems to be similar to that found in the teeth of an adult diplodocids (CM 11161, Fig. 7). In the titanosaurian *Lirainosaurus* differences are also observed between juvenile teeth, with smooth enamel and cylindrical cross section, and adult teeth, described as D-shaped in cross-section and with ‘more ornamented enamel’ wrinkling [88]. The enamel of the teeth of the Auca Mahuevo titanosaurian embryos is also reported to be smooth, whilst adult titanosaurs display wrinkling [89,90]. Also, the enamel/dentine ratio (total enamel surface area/total tooth area) differs between ontogenetic stages, with a higher enamel/dentine ratio for embryos than for adult titanosaurs, and the embryonic teeth lack lingual curvature as opposed to adults [93].

In summary, the wrinkling pattern of the tooth enamel of neosauropods and macronarians does not seem to change during ontogeny, with the exception of titanosaurs. This finding supports the hypothesis that the division of our sample into several morphotypes based on enamel wrinkling probably reflects taxonomic variation, even though special care must be taken when the studied sample includes teeth of significantly different sizes (which may represent different ontogenetic stages). This, however, is not the case of the sample of isolated teeth studied in this contribution.

### Taxonomic variation

The craniomandibular material of morphotype I and II (dentaries MACN-CH 933, MPEF-PV 1670, and maxillae MACN-CH 934) were all assigned to *Patagosaurus* [21,23]. However, in this study and in previous work [23] significant differences are found in tooth morphology between the dentaries MACN-CH 933, MPEF-PV 1670 and the maxilla MACN-CH 934. Even though differences between the maxillary dentition and that of the dentary are not uncommon in sauropods (as mentioned before), in this case the postcranial material associated with the maxillae of MACN-CH 934 is found to be different from the postcranial material associated with MACN-CH 933, which in turn is found to overlap with that of the holotype of *Patagosaurus* [21,23]. This indicates that MACN-CH 934 is unlikely to represent *Patagosaurus* [23].

Furthermore, MACN-CH 933 and MPEF-PV 1670 are comparable not only in enamel wrinkling but also in general dentary morphology [23]. Since these teeth of morphotype I are assigned to *Patagosaurus* (see Table 1), as associated cranial material (although not holotype material), we can tentatively refer morphotype I to *Patagosaurus fariasi*. This assignment should, however, be seen as tentative, as there is no cranial or dental material directly associated with the holotype.

Thus, apart from differences in postcranial morphology [22,23] this study demonstrates that the dentition of MACN-CH 934 also indicates that this specimen differs from the holotype of *Patagosaurus* and referred material. Therefore, MACN-CH 934, including the maxilla with teeth of morphotype II, may belong to an undescribed sauropod taxon, other than *Patagosaurus*. As already noted by Rauhut [23], the material referred to *Patagosaurus* is in need of revision. Morphotype III belongs to yet another undescribed sauropod, which was first reported by Pol et al. [25]. All of these morphotypes can be correlated with postcranial material, and a further taxon, *Volkheimeria*, is so far represented by postcranial material only [21]. Thus, the sauropod diversity of the Cañadón Asfalto Fm. is higher than previously assumed, with at least four different taxa being present. The further confirmation that the material originally referred to *Patagosaurus* includes a different taxon of eusauropod makes a revision of all of this material necessary; such a revision will greatly aid in the study of the early evolution and radiation of eusauropods in the Middle Jurassic.

The possibility that morphotype I, which accounts for the isolated teeth, represents more than one taxon cannot be ruled out completely. Since *Volkheimeria* is known from only one partial skeleton with no cranial material, some of these teeth might represent that taxon. However, given that *Patagosaurus* is known from abundant material, the chance is higher that our sample does not contain *Volkheimeria* teeth. More material is needed in order to rule out either option.

## Conclusions

Based on observations of the eusauropod toothrows and isolated teeth from the Cañadón Asfalto Formation (MPEF-PV 3341, MACN-CH 933, MPEF-PV 1670, MACN-CH 934), as well as from other taxa (e.g., *Camarasaurus*, diplodocids, *Giraffatitan*), it seems that enamel wrinkling pattern is conservative along the toothrow in sauropods, (including basal eusauropods, diplodocids, and macronarians), so that there is minimal intraspecific variation, but considerable interspecific variation. This indicates that enamel wrinkling may be used as a valid character for morphotype definition and can provide information about sauropod diversity in poorly sampled faunas. However, caution should be taken with ontogenetic variation as there are reported cases (thus far only of titanosaurs) in which enamel wrinkling and enamel thickness varies along ontogeny [88,93].

We used enamel wrinkling pattern as a primary character to categorize isolated teeth and craniomandibular teeth from different localities from the Cañadón Asfalto Formation into three distinct morphotypes. Within the material originally assigned to *Patagosaurus* we could distinguish two different morphotypes, supporting the claim that more than one taxon is represented by this material [23]. A third morphotype represents a so far undescribed eusauropod taxon from the El Bagual locality [25]. The similar outcome of eusauropod diversity based on tooth morphology and postcranial anatomy indicates the usefulness of studies of isolated teeth for basic taxonomical purposes, such as diversity estimates. This implies that 1. at least three different sauropod species with known dentition are recorded in the Cañadón Asfalto Formation (*Volkheimeria* being the only eusauropod taxon from that unit without known dentition), and that 2. the two undescribed eusauropod taxa represented by MACN-CH 934 and the El Bagual locality can be distinguished from *Patagosaurus* based on their tooth morphology.
